# Evaluation of Newly Developed Easy-Open Assistive Devices for Pneumatic Tube System Carriers for the Reduction of Work-Related Musculoskeletal Disorders

**DOI:** 10.1155/2021/8853602

**Published:** 2021-01-08

**Authors:** Tzu-I Chien, Huey-Wen Liang, Ya-Fen Lee, Fei-Yun Liu, Chi-Kwang Hsu, Shao-Tseng Liu, Mo Siu-Mei Lee, Pin-Fei Wei

**Affiliations:** ^1^Department of Laboratory Medicine, National Taiwan University Cancer Center, Taipei, Taiwan; ^2^Department of Physical Medicine and Rehabilitation, National Taiwan University Hospital and College of Medicine, Taipei, Taiwan; ^3^Department of Laboratory Medicine, National Taiwan University Hospital, Taipei, Taiwan; ^4^Department of Biomedical Engineering, National Taiwan University Hospital, Taipei, Taiwan; ^5^Department of Mechanical Engineering, National Taiwan University, Taipei, Taiwan; ^6^Department of Laboratory Medicine, National Taiwan University Hospital and China Medical Hsinchu University Hospital, Taipei, Taiwan

## Abstract

Musculoskeletal disorders may affect labor efficiency, cause disability, impair one's work ability, and lower one's quality of life. This consequently leads to a larger expenditure of medical resources. We aimed to design easy-to-open assistive devices for pneumatic tube systems to improve ergonomics and reduce musculoskeletal complaints of workers. We followed a design control process, including designs of motors, gears, sensors, and V-shaped connecting rods. Efficacy was evaluated by examining risks based on job strain index, user satisfaction, and musculoskeletal complaints of operators before and after the system's implementation on a Nordic musculoskeletal questionnaire. We designed three assistive devices: two semiautomatic and one automatic. Each semiautomatic device costs about 300 US dollars and required space of 10 × 18 × 38 cm^3^. The automatic device costs about 3000 US dollars and required space of 28 × 38 × 50 cm^3^. The job strain index score decreased from 36 (very high risk) to 3 (low risk) with the semiautomatic devices and to 0 with the automatic device. Musculoskeletal complaints in the neck and upper limbs were reduced, with a significantly higher satisfaction rate for female operators. Our novel design of an automatic cap opening device for a pneumatic tube system was effective in improving ergonomics and reducing musculoskeletal complaints.

## 1. Introduction

Work-related musculoskeletal disorders (WRMSDs) are common health problems, and the number of WRMSD cases is increasing in workplaces [1]. Occupational injuries caused by repetitive motion have resulted in workplace absences with a median of nine working days per year, costing industries US$ 13–20 billion per year [2]. According to the Occupational Safety and Health Statistics by the Ministry of Labor of Taiwan, about 40% of WRMSDs are work-related [3]. These injuries can be caused by overexertion, awkward work posture, repetitive work, or a lack of proper rest. The number of cases of injury to the arm, shoulder, and neck has been growing significantly, accounting for the largest proportion of labor insurance payments [1]. Musculoskeletal disorders may affect labor efficiency, cause disability, impair one's work ability, and lower one's quality of life. This can consequently lead to a larger expenditure of medical resources. For these reasons, the prevention of WRMSDs has become an important issue for occupational health and safety.

Previous studies have shown that laboratory personnel who use pipettes have elevated risk factors for carpal tunnel syndrome [4]. A review of WRMSDs has been undertaken among medical laboratory professionals [5]. A review of nonpowered hand tool improvement research indicated that the orientation of hand locations also causes various health-related problems [6]. Repetitive, hand-intensive movements contribute to the development of hand/wrist WRMSDs [7]. Studies have reported that opening processes can be improved using human factors engineering [8, 9].

A pneumatic tube system (PTS) has been the conventional method of transporting medical records, medicines, specimens, or blood bags for many years. In a hospital with 2700 beds, the number of daily transports can reach 1600 and may exceed 680,000 times per year. The PTS transports goods up to 5 kg in weight in a carrier through a tube network within the whole hospital, across long distances, and to all floors. The use of the PTS as a transport tool can significantly shorten transport times and reduce labor costs. Nevertheless, the repeated movement associated with opening the cap with an overly opened palm arc often causes cumulative musculoskeletal occupational disorders. The new 6-inch (diameter: 16 cm) pneumatic tube carrier (Swisslog TranspoNet system, Westerstede, Germany) can transport a larger volume of products than the old 4-inch device. The increased diameter and weight of the pneumatic tube carrier cap make it harder for operators with smaller hands to open the cap. Generally, pneumatic tube carriers are circular, smooth, and wide in diameter and lack a fulcrum point. When operators open the cap with an overly opened palm arc, the force of the torque increases, facilitating repeated movements, often causing cumulative musculoskeletal occupational disorders, as mentioned by Gerr et al. [10].

This study applied the design control process [11] as a framework, and we referred to a document called “Applying Human Factors and Usability Engineering to Optimize Medical Device Design and Design Control Guidance for Medical Device Manufacturer” [11–15]. The Food and Drug Administration's Design Control process includes user research (observation and interview), as well as clinical, technical, and business requirements. During the daily work routine, we found that operating the PTS may create and aggravate disorders of the hands of the operators. First, we identified the cause by conducting a general survey of musculoskeletal occupational disorders.

Previous studies showed that assistive devices can help people with severe disabilities and patients with hand disorders to open jars [8, 9]. The goal of this study aligned with the concepts of ergonomics—obtaining a good match between the worker and the job. Using human factors engineering and usability, we conducted an overall assessment for validation and verification during various stages of development and the implementation of a novel user-friendly automatic cap opening device for pneumatic tube carriers. We aimed to design easy-to-open assistive devices for pneumatic tube systems (PTSs) to improve ergonomics and reduce the musculoskeletal complaints of workers.

## 2. Materials and Methods

**Figure 1:**
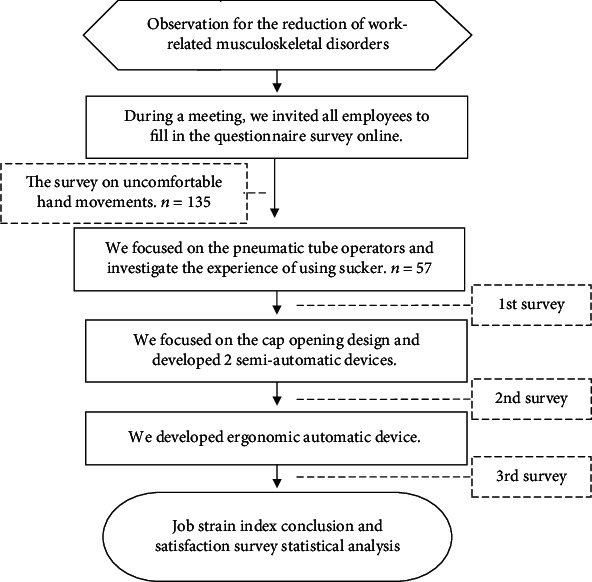
Flowchart.

In this study, we first conducted a general survey about musculoskeletal occupational disorders to identify the task that could cause such disorder; then, we applied the waterfall model in developing assistive devices for the said task. During the developing process, a satisfaction survey and JSI assessment were conducted for each generation of devices. The flow chart is shown in Figure 1.

### 2.1. Subjects

The protocol of this study is in accord with the Helsinki Declaration of 1975, as revised in 2008, and was approved by the institutional review board of the National Taiwan University Hospital (approval number 201805101W). Responding to the questionnaire was voluntary and considered consent for participation since 2015 to 2019. During a meeting, we invited all 181 employees at the Department of Laboratory Medicine of National Taiwan University Hospital to fill in the survey online voluntarily. A total of 135 employees responded, and 57 of those employees who operated the PTS took part in the remainder of the study. Of these 57 participants, 38.6% were male and 61.4% were female and the age range was 20–59 years. The years of experience was 3–10 years, the working hours were 8 hours per day, and most participants were right-handed.

After production, the assistive devices were given to the operators to use in their daily work routines.

### 2.2. Methods

#### 2.2.1. The Design Evolution Process

We first conducted a general survey of musculoskeletal occupational disorders to identify tasks that could cause such disorders and then we applied the waterfall model [16, 17] to develop assistive devices for these tasks. During the developing process, a satisfaction survey and strain index (SI) assessment were administered for each generation of devices. The design process also included the following steps: (a) literature review of pneumatic tube carriers, WRMSDs, and ergonomics; (b) opinions of the users from the hospital; (c) competitive product benchmarking; (d) concept development and ergonomic testing; (e) user testing and feedback; and (f) product evolution and improvement.

### 2.3. Survey

Three surveys were administered in this study. The data were collected from a training management system and field questionnaires with the design of assistive devices. Each survey included close-ended questions about musculoskeletal symptoms (Nordic musculoskeletal questionnaire), satisfaction degree (5-point Likert scale) on the new opening process, preference for the assistive devices, and open-ended questions on reasons and suggestions. The survey items are listed in the Table 1.

The interval between each survey was approximately 1 year ± 1 month due to the research and development process. The same demonstrator trained operators in using the device in groups of approximately five, and then, the operators filled in the survey within two weeks.

#### 2.3.1. Feedback Loops between the Processes and Outcome Design

Global regulations provided a standard method to design and develop the device based on the needs and requirements of the users [15, 16]. The assistive devices were designed under the consideration of occupational environments and user requirements. We analyzed the hand movements and exertion when opening the caps and developed customized, innovative, assistive devices that fitted the needs of the operators.

### 2.4. Ergonomic Risk Assessment

The job strain index (JSI) was applied to evaluate hand/wrist postural risks to the worker. Three operators perform 3 cycles. Videos were played forward and backward by 2 rehabilitation therapists to reach a consensus and scrutinize six factors: the intensity and duration of exertion, efforts/minute, hand/wrist posture, the speed of work, and duration per day. The JSI was determined by adding scores for these six factors. A JSI assessment was carried out each time an accessory was completed. The risk was divided into four levels: (a) lower than 3 as safe; (b) 3 to 5 as uncertain; (c) 6 to 7 as some risk; and (d) higher than 7 as hazardous according to various index values for each item [15].

#### 2.4.1. Measures of Pushing Force

We used the DBU-120A test procedure instead of hand torque during the opening phase, whereby the DBU-120A controller (Kyowa Electronic Instruments Co., Tokyo, Japan) and a computer were connected and parameters configured, and thereafter, the operating status of the controller was monitored.

### 2.5. Statistical Analyses

In the symptoms of physical pain in the Nordic musculoskeletal questionnaire, we use the Pearson chi-square test. The alpha level was set at 0.05. In the satisfaction survey, we used a 5-point Likert scale rating with the following ranges: 1 = very unsatisfied, 2 = unsatisfied, 3 = fair, 4 = satisfied, and 5 = very satisfied. The differences among the five operation methods, hand, sucker, push-open, rotate-open, and automatic cap opening device, were compared using Fisher's exact test. These operation methods were trialed across the same participants. Satisfaction rate = numerator (very satisfied + satisfied)/denominator (gender). The difference between males and females was assessed with a Bonferroni correction for multiple comparisons. All data analyses were performed using SAS 9.4 (SAS Institute Inc., Cary, NC, USA).

## 3. Results

### 3.1. Observation

We found that repetitive opening and no force support points were risk factors. The assistive devices should be designed and revised from the user's point of view following the design control process. The predesign survey results from 135 participants and the 57 participants who operate PTS in this study are shown in Supplementary Table S1.

**Figure 2:**
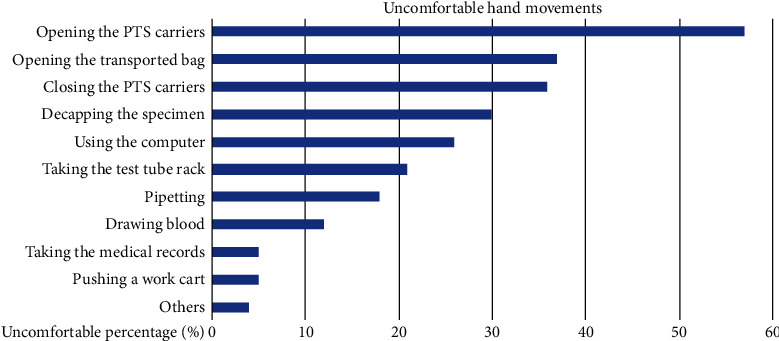
Results of the survey on uncomfortable hand movements.

The questionnaire results indicated that 57% of participants believed that one of the reasons for hand discomfort was opening the PTS carriers and 36% believed it was due to closing the PTS carriers (Figure 2). This result formed the motivation for our research. A focus group was formed comprising PTS operators. We used the Nordic musculoskeletal questionnaire to survey the use of assistive devices for the PTS. We conducted interviews and efficacy assessments for the cap opening device design. We considered the special requirements of the device including transportation of samples, blood bags, and medicines between the Department of Medical Records, the pharmacy, the blood banks, nursing stations, and the Department of Laboratory Medicine. Responses included the following: “After using the computer for a prolonged period of time, the hands of operators are already in pain, and opening PTS caps make the pain even worse.” and “For those with smaller hands, it is very laborious to open caps. Especially, the new PTS is tighter and more difficult to operate.”

### 3.2. Ergonomic Devices

**Figure 3:**
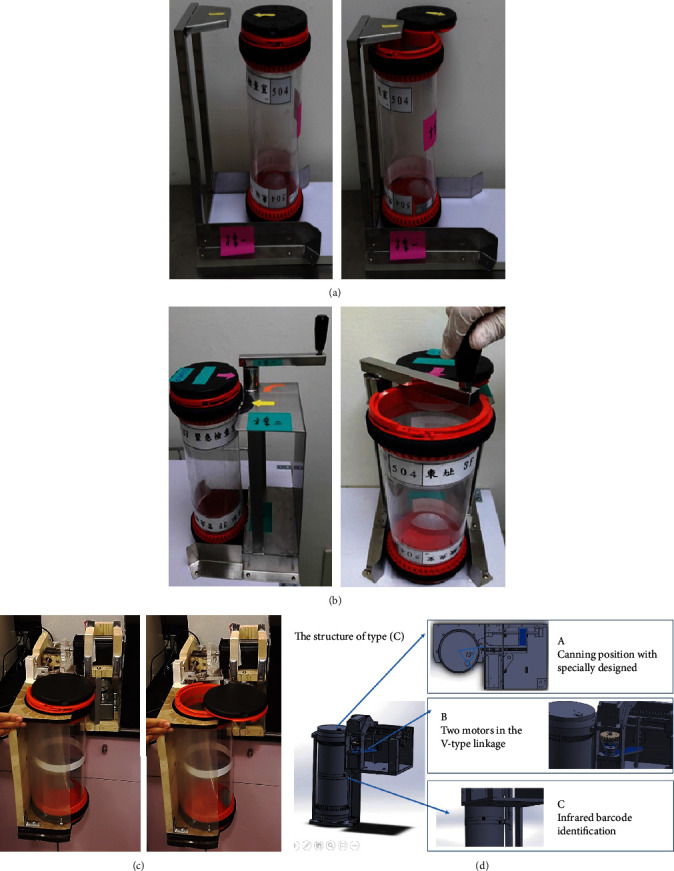
Three newly developed assistive devices, including (a) a push-open semiautomatic assistive device, (b) a rotate-open semiautomatic assistive device, and (c) a cap opening automatic device. (d) The structure of type (C): (a) canning position with the bottle cap shaft and specially designed open can pusher; (b) all work is done using only two motors in the V-type linkage design; (c) infrared barcode identification can opening position design; user self-completion of rapid barcode installation.

We looked for a commercially available product suction cup that could be used to open the carrier. Then, we evaluated three different newly developed assistive devices, including semiautomatic assistive devices named as follows: (A) push-open, (B) rotate-open, and (C) automatic cap opening devices (Figure 3). The structures of (A) and (B) included a rail in the front; they were made of stainless steel and were tailored to the height and diameter of the pneumatic tube carriers. Type (A) pushed the carriers forward, which could reduce the effort needed to push the lid away. Type (B) pushed the carriers along the rail to a fixed point with a rotational effort to open the cover. The structure of type (C) included the open position and cap shaft by a specially designed open pusher. The V-shaped connecting rods were designed to complete the work using two motors and gear wheels. We designed an infrared barcode scanner to open the cap, and users could quickly complete the barcode installation themselves. The assistive devices were tested by the operators, and a satisfaction survey was administered. The PTS carrier was placed and automatically sensed by infrared barcode positioning, while the pushrod completed the open cover (Supplementary video (available here)).

### 3.3. Musculoskeletal Symptoms

**Figure 4:**
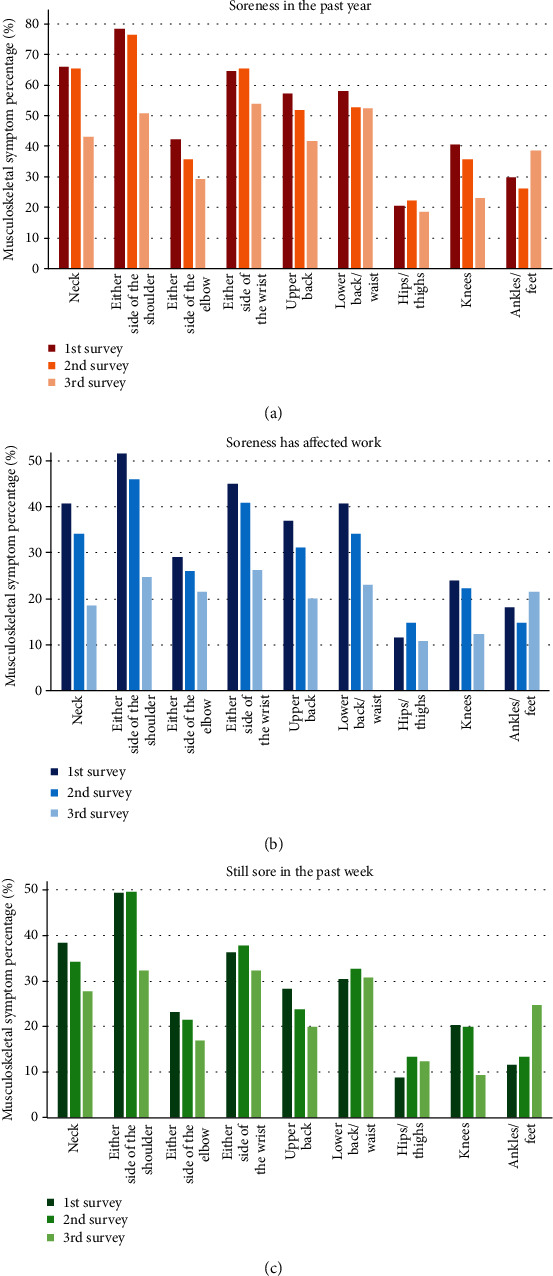
Musculoskeletal symptoms according to the number of cases with soreness in the past year (a), cases where the soreness has affected work (b), and cases that were still sore in the past week (c).

The Nordic musculoskeletal questionnaire is included in the Table 1. Pain in the wrist/hand (64.5%) was second only to shoulder (78.3%) and neck pain (65.9%) in the musculoskeletal questionnaire.  Figure 4 shows the number of cases with soreness in the past year, the number of cases where the soreness has affected work, and cases still sore in the past week. After the improvement of assistive devices, the rate of shoulder discomfort had dropped from 78.3% to 50.7%, and the soreness that affects work had also dropped from 51.5% to 24.6%. The wrist/hand complaints had dropped from 64.5% to 53.9%, and the soreness that affects work had also dropped from 44.9% to 26.2%. There was a tendency of the reduction of soreness after 3 years, especially in the upper limbs (Figure 4).

### 3.4. Satisfaction Survey

**Figure 5:**
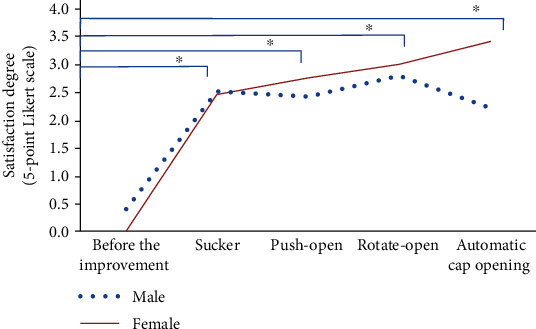
Operator satisfaction survey. ^∗^Calculated using Fisher's exact test (*P* < 0.0001).

The satisfaction survey showed that all improvement devices were better than the original bare-handed opening cover (*P* < 0.0001) (Figure 5). The operator satisfaction survey showed that satisfaction was excellent and improved from the sucker device to the semiautomatic (push-open and rotate-open device) to the automatic easy-open assistive device. The satisfaction rate increased from 0 to 67.9% in female. The rate of “fair” satisfaction dropped to zero with the use of the automatic easy-open assistive device (data not shown). Satisfaction was significantly higher in females because of their smaller hand size. The improvement was amplified further by the female-dominated sex ratio, which was approximately 1.6 : 1 in this study.

### 3.5. Job Strain Index

**Figure 6:**
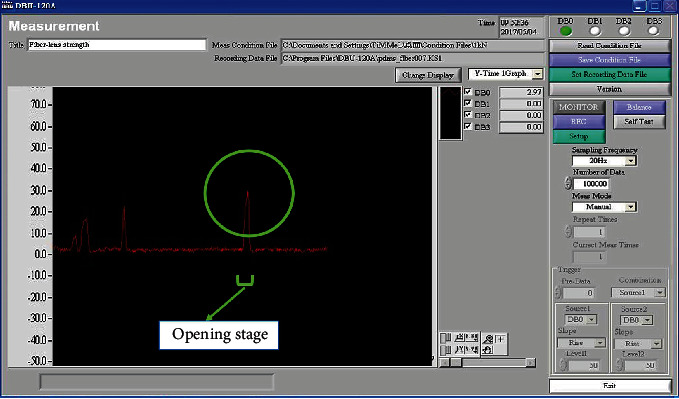
The force required to use the DBU-120A test program instead of hand torque during the opening phase is approximately 150 N.

**Figure 7:**
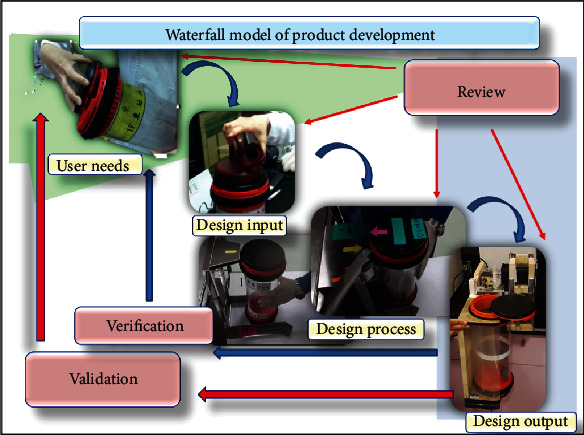
The waterfall model of our medical device innovation process map.

The JSI was calculated by multiplying six factors. Decomposition of the strain index was calculated for various cap opening movements (Supplementary Table S2). Using freehand opening (pretrial), the JSI total score was 36 (extremely high risk, need to improve immediately). This decreased significantly to 3 for the use of push-open and to 1.5 for the use of rotate-open devices (low risk, safe work). Importantly, the risk score for the automatic easy-open assistive device dropped to zero. This suggests a much higher efficacy for our newly developed assistive devices (Supplementary Table S3). The force required to use the DBU-120A test program instead of hand torque during the opening phase was approximately 150 N (Figure 6). The waterfall model of our medical device innovation process map is shown in Figure 7.

### 3.6. Cost-Effectiveness

We could not find any appropriate easy-open devices after searching for all commercial products. To build a device for automatic unloading of PTS, the cost would be at least US$ 65,000. The required space for the original system was approximately 70 × 80 × 220 cm^3^, and the original system could not transport blood bags. The innovative semiautomatic assistive devices developed in this study cost about US$ 300, and the required space would be 10 × 18 × 38 cm^3^. The automatic assistive devices cost about US$ 3000, and the required space would be 28 × 38 × 50 cm^3^. Our newly developed devices could be customized according to the size of the pneumatic tube carriers. The devices are suitable for different brands and suitable for the transport of blood bags, specimens, medicines, and medical records.

## 4. Discussion

We determined that the main cause of soreness with the use of PTS carriers was associated with the opening and closing of the carrier. Our search for a suitable commercially available product was not successful because the appropriate product required more specialized accessories, and there are several steps involved in the use of a PTS carrier. While we found automatic unloading pneumatic tubes, the cost was high, and the space required was too large (as mentioned in Cost-Effectiveness), rendering installation in general hospitals difficult. Therefore, we adopted the steps of a design control process for auxiliary research and development, and each process step was accompanied by a questionnaire to collect feedback from users. We assessed the functionality of our semiautomatic device, and this allowed us to optimize the development of a fully automatic auxiliary, with the third survey (on the fully automatic device) showing a hazard factor of 0. This result was also reflected in the satisfaction survey, with an average 13-fold increase in satisfaction and an improvement in upper limb discomfort. Musculoskeletal symptoms on the shoulder and wrist/hand decreased 52.2% and 41.8%, respectively, in the soreness that has affected work.

In recent years, musculoskeletal disease among workers in various countries has emerged as one of the most serious occupational health problems. Previous studies have found that the risk factors associated with musculoskeletal disease in the upper extremities were repeated hand movements, unnatural work postures, the use of vibrating hand tools, higher work pressures, and longer work hours [17]. Reports have shown that work posture or exercise may affect the occurrence of musculoskeletal disease in the upper extremities [5]. One study showed that musculoskeletal disease occurred in technicians using pipettes [18]; studies have focused on reducing the incidence of WRMSD [4, 19]. Other studies focused on medical staff who experience physical discomfort during surgery [20] and the wrist or hand pain experienced by hospital-based nurses [21] using the JSI for the assessment of musculoskeletal risk factors [22]. Many other studies have used the JSI assessment of musculoskeletal risk factors [23], and others have analyzed upper limb disease risk [24] and robotic-assisted surgery for ergonomics in surgery [25]. These studies echo our original intention to improve the incidence of WRMSD.

Operators with smaller palms and joint-related diseases had significantly improved usage and improved work efficiency with the use of the accessories used in this study. However, it is inevitable that there are blind spots in the development of medical equipment. Even if a problem does not manifest itself in the general population during testing, this does not mean that people with slightly larger or smaller hands compared to the average will not experience discomfort. Furthermore, we cannot assume that there will not be problems, as long-term use could still cause harm. Consideration should be given to the use of sensitive groups (small palms and related diseases) during product testing to ensure that the product does not burden the user.

This study had several limitations. First, it was based on questionnaires; therefore, response bias needs to be considered. The extent of discomfort may be underreported because employees with hand injuries might have opted out of this workstation, such as those with carpal tunnel syndrome or arthritis. Second, our device was not tailored for different hand sizes.

### 4.1. Application of Accessories

The market for our design concept can expand beyond medical equipment, such as medicine cans and canned food. The design of this accessory was not restricted by the factory, such as Sumetzberger (ROBO System, Sumetzberger, Austria) or Aerocom (Aerocom Limited, UK), but rather involves only a different way of opening the cover (model restrictions).

## 5. Conclusions

In conclusion, we encountered high-frequency repetitions and unnatural dangerous postures in the workstation and made improvements. Our devices were able to meet ergonomic standards more effectively than existing PTS devices (as shown in Figure 7). Our next step will be to seamlessly connect our automatic device to several PTS outlets. We hope that through collaboration between different departments, we can prevent occupational injuries and improve the workplace environment.

## Figures and Tables

**Table 1:** Nordic musculoskeletal questionnaire. You need to assist with the musculoskeletal symptoms questionnaire to improve your working environment.

Category	Options						
Your gender:	Male	Female					

Your age:	20–29 years old	30–39 years old	40–49 years old	50–59 years old	≥60 years old		

Which hand do you usually use?	Right	Left					

Average of hours worked: hours/day	4 hours/day	8 hours/day	9 hours/day	10 hours/day	12 hours/day		

Your total years of work in our hospital: years	0 to 2	3 to 5	6 to 10	11 to 15	16 to 20	>20	

Workplace Safety							

In the last six months, do you have the most obvious hand/wrist discomfort at work?	Taking the test tube rack	Opening the PTS carriers	Closing the PTS carriers	Opening the transported bag	Using the computer	Drawing blood	Pipetting

In the last six months, do you have the most obvious hand/wrist discomfort at work?	Pushing a work car	Decapping the specimen	Taking the medical records	Others			

Do you need to use PTS at work in the past six months?	Yes (please continue with the following questions)		No (please skip the question, physical pain symptoms, other items are not applicable)				

The approximate frequency at which you perform the PTS per day	25 times	25 to 49 times	50 to 99 times	100 to 149 times	150 to 200 times	Other	N/A

Have you ever experienced the following problems when you are performing the PTS carrier? (Multiple choice)	The PTS carrier is not easy to open	Hard to apply	Hand damage	Feel the PTS carrier is too heavy	Others	N/A	

How satisfied are you with the past use of the PTS (small PTS carrier)?	Very satisfied	Satisfied	Fair	Unsatisfied	Very unsatisfied	N/A	

How satisfied are you with the current use of the PTS (large PTS carrier)?	Very satisfied	Satisfied	Fair	Unsatisfied	Very unsatisfied	N/A	

Have the following problems been improved after improving the PTS mode? (Please check with improvement questions)	The PTS carrier is not easy to open	Hand force is not easy	Hand damage	No	N/A	Other	

What is your satisfaction with the current improvement?	Very satisfied	Satisfied	Fair	Unsatisfied	Very unsatisfied	N/A	

Symptoms of physical pain	★ the following questions to ask about your recent physical pain. ★ the term "pain" in the question means: For discomforts such as soreness, tenderness, tingling, or numbness that last more than a day, please read the descriptions carefully and recall their answers, and if there is no pain, click in the "O" in front of "No".						

Neck							

Have you ever had pain or soreness or discomfort in your neck in the past year?	None (Please skip the question, shoulder, other items are not applicable)				Yes (please continue with the following questions)		

Did pain in the neck area affect your normal life or work over the past year?	No	Yes	N/A				

Have you had any pain in your neck in the past week?	No	Yes	N/A				

Shoulder							

Have you ever had pain or soreness or discomfort in your shoulders in the past year?	None (Please skip the question, elbow, other items are not applicable)				Yes, on both sides.	Yes, left.	Yes, right.

Did pain in the shoulder area affect your normal life or work over the past year?	No	Yes	N/A				

Have you also had pain in your shoulder in the past week?	No	Yes	N/A				

Elbow							

Have you ever had pain or soreness or discomfort in the elbow position in the past year?	None (Please skip the question, wrist/hand, other items are not applicable)				Yes, on both sides.	Yes, left.	Yes, right.

Did pain in the elbows affect your normal life or work over the past year?	No	Yes	N/A				

Have you had any pain in your elbow in the past week?	No	Yes	N/A				

Wrist/Hand							

Have you ever had pain or soreness or discomfort in your wrist/hand in the past year?	None (Please skip the question, upper back, other items are not applicable)				Yes, on both sides.	Yes, left.	Yes, right.

Did pain in the wrist/hand area affect your normal life or work in the past year?	No	Yes	N/A				

Do you have any pain in your wrist/hand in the past week?	No	Yes	N/A				

Upper back							

Have you ever had pain or soreness or discomfort in your upper back in the past year?	None (Please skip the question, lower back/waist, other items are not applicable)					Yes	

Did upper back pain affect your normal life or work over the past year?	No	Yes	N/A				

Have you had pain in your upper back in the past week?	No	Yes	N/A				

Lower back/waist							

Have you ever had lower back/lower back pain or soreness or discomfort in the past year?	None (Please skip the question, hip/thigh, other items are not applicable)					Yes	

Did lower back/lower back pain affect your normal life or work during the past year?	No	Yes	N/A				

Have you had lower back/lower back pain in the past week?	No	Yes	N/A				

Hips/Thighs							

Have you ever had hip/thigh pain or soreness or discomfort in the past year?	None (Please skip the question, knee, other items are not applicable)					Yes	

Did hip/thigh pain affect your normal life or work during the past year?	No	Yes	N/A				

Do you have hip/thigh pain in the past week?	No	Yes	N/A				

Knees							

Have you ever had knee pain or soreness or discomfort in the past year?	None (Please skip the question, ankle/foot, other items are not applicable)					Yes

Did knee pain affect your normal life or work over the past year?	No	Yes	N/A				

Have you had knee pain in the past week?	No	Yes	N/A				

Ankles/Feet							

Have you ever had ankle/foot pain or soreness or discomfort in the past year?	None (Please skip the question, wrist/hand symptom, other items are not applicable)					Yes	

Did ankle/foot pain affect your normal life or work over the past year?	No	Yes	N/A				

Do you have ankle/foot pain in the past week?	No	Yes	N/A				

Wrist/Hand Symptoms							

Have you ever had to change work or daily activities because of hand/wrist pain?	No	Yes					

How long has your hand/wrist pain lasted in the past year?	0 days	1 to 7 days	8 to 30 days	More than 30 days, but not every day pain	There's pain every day.		

Would you need to reduce work or housework in the past year because of hand/wrist pain?	No	Yes					

Do you need to reduce leisure activities in the past year due to hand/wrist pain?	No	Yes					

How much time have you spent in the past year unable to do work or housework because of hand/wrist pain?	0 days	1 to 7 days	8 to 30 days	More than 30 days			

Have you ever needed medical attention in the past year because of hand/wrist pain?	No	Yes					

Taken together, your recommendations are:							
Thank you for your advice!							

## Data Availability

The data used to support the findings of this study are available from the corresponding author upon request.

## Supplementary Materials

Supplementary Materials Supplementary Table S1: participant characteristics. Supplementary Table S2: decomposition of the strain index was calculated for various cap opening movements. Supplementary Table S3: checklist for various newly developed assistive devices by job strain index. Supplementary video: the automatic easy-open assistive devices for PTS carriers.
