# Supplementary material for: Evaluation of Newly Developed Easy-Open Assistive Devices for Pneumatic Tube System Carriers for the Reduction of Work-Related Musculoskeletal Disorders
Source: Biomed Res Int. 2021 Jan 8;2021:8853602. doi: 10.1155/2021/8853602 (PMC7810564; doi:10.1155/2021/8853602)
Supplement: Supplementary Materials — Supplementary Table S1: participant characteristics. Supplementary Table S2: decomposition of the strain index was calculated for various cap opening movements. Supplementary Table S3: checklist for various newly developed assistive devices by job strain index. Supplementary video: the automatic easy-open assistive devices for PTS carriers. [file 8853602.f1.docx]

**Supplementary Table S1**. Participant characteristics

|  |  | pre-R&D  General survey* | | post-R&D  Satisfaction survey** | | |
| --- | --- | --- | --- | --- | --- | --- |
|  |  | **Number** | **%** |  | **Number** | **%** |
| Gender | | | | | | |
|  | Male | 41 | 30.4 |  | 22 | 38.6 |
|  | Female | 94 | 69.6 |  | 35 | 61.4 |
| Age categories | | | | | | |
|  | 20–29 y | 33 | 24.4 |  | 21 | 36.8 |
|  | 30–39 y | 31 | 23.0 |  | 16 | 28.1 |
|  | 40–49 y | 47 | 34.8 |  | 13 | 22.8 |
|  | 50–59 y | 22 | 16.3 |  | 7 | 12.3 |
|  | > 60 y | 2 | 1.5 |  | 0 | 0.0 |
| Seniority*** | | | | | | |
|  | 0–2 y | 26 | 19.3 |  | 17 | 29.8 |
|  | 3–10 y | 38 | 28.1 |  | 21 | 36.8 |
|  | 11–20 y | 37 | 27.4 |  | 8 | 14.0 |
|  | >20 y | 34 | 25.2 |  | 11 | 19.3 |
| Working hours | | | | | | |
|  | 8 h | 98 | 72.6 |  | 44 | 77.2 |
|  | 9-11 h | 35 | 25.9 |  | 12 | 21.1 |
|  | 12 h | 2 | 1.5 |  | 1 | 1.8 |
| Handedness | | | | | | |
|  | Right | 131 | 97.0 |  | 56 | 98.2 |
|  | Left | 4 | 3.0 |  | 1 | 1.8 |

*A general survey about musculoskeletal occupational disorders (135 participants)
**A satisfaction survey about the newly developed assistive devices for pneumatic tube system users (57 participants)

***Working years

**Supplementary Table S2**. Decomposition of strain index was calculated for various cap open movements.

(A) Decomposition of strain index was calculated for push-open assistive devices.

| action | set up push-open assistive devices | force opening | pickup specimens | close the cap |
| --- | --- | --- | --- | --- |
| process photos | 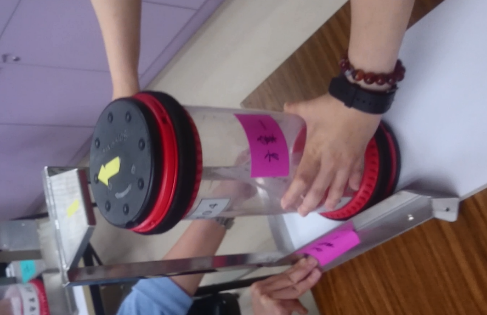 | 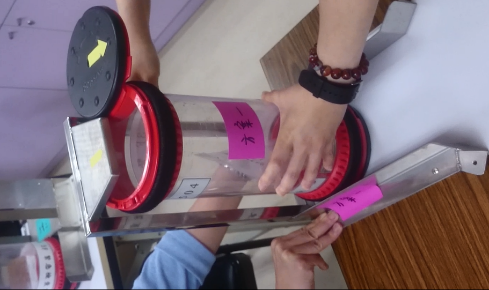 | 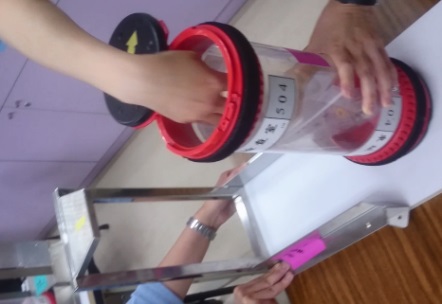 | 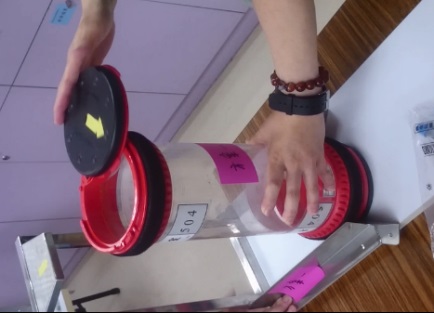 |
| action time | 2 seconds | 2 seconds | 2 seconds | 1 second |
| intensity of exertion | 1 | 2 | 1 | 1 |

(B) Decomposition of strain index was calculated for rotate-open assistive devices.

| action | set up rotate-open assistive devices | force opening | pickup specimens | close the cap |
| --- | --- | --- | --- | --- |
| process photos | 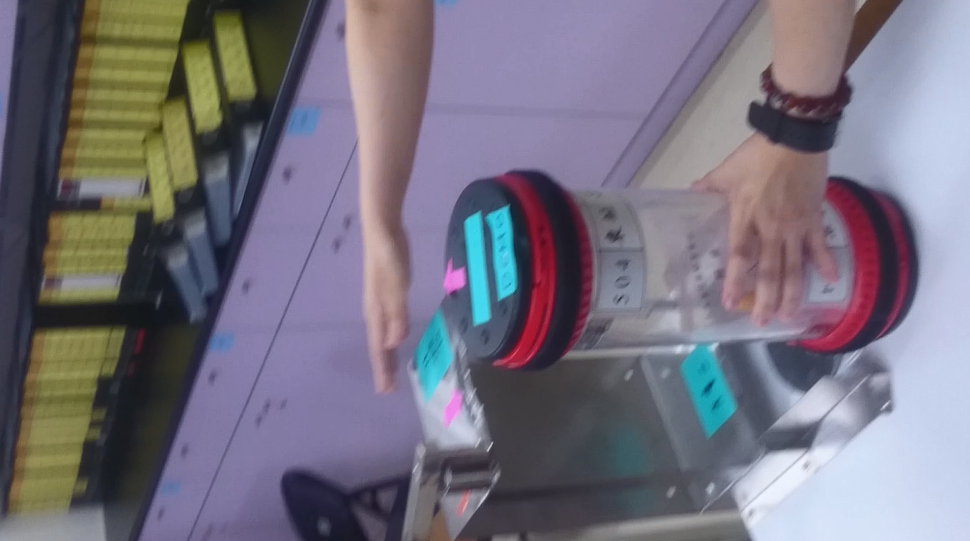 | 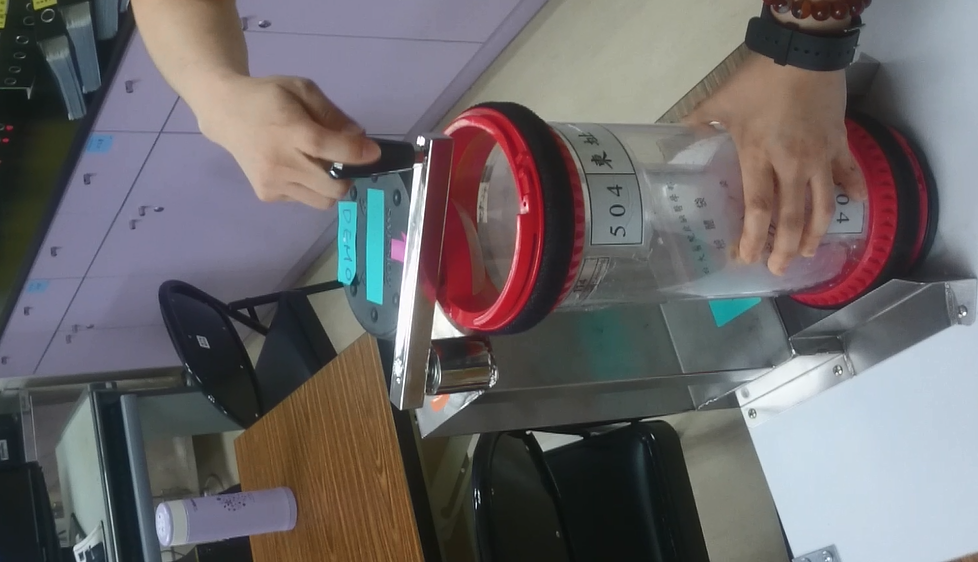 | 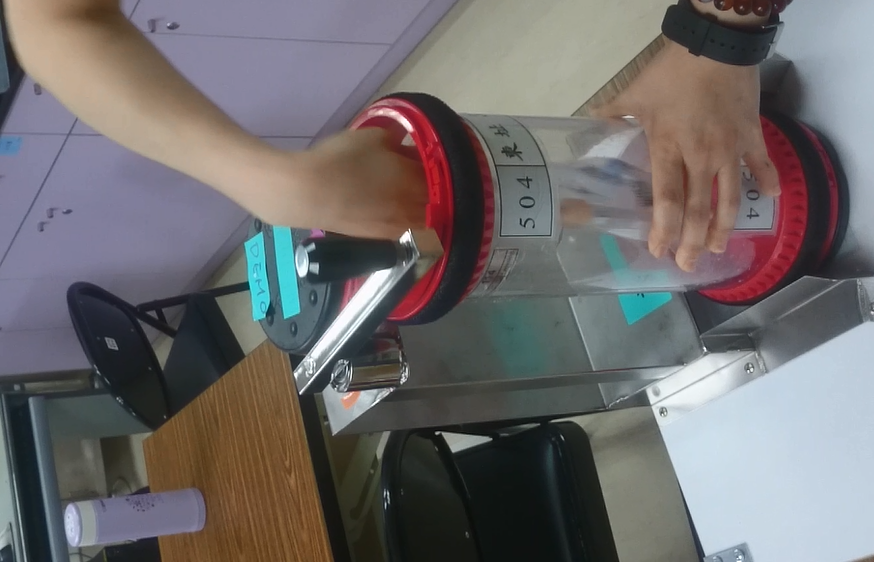 | 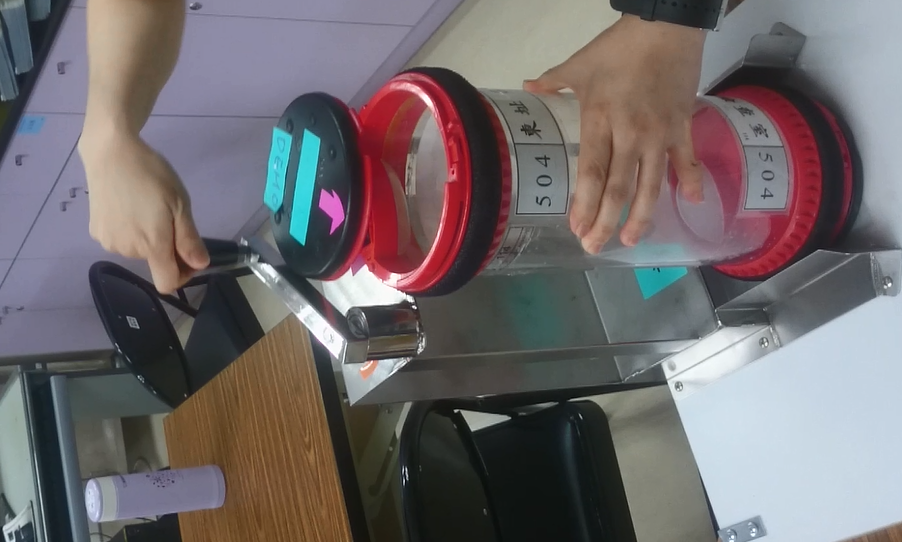 |
| action time | 5 seconds | 1 second | 2 seconds | 1 second |
| intensity of exertion | 0.5 | 1 | 1 | 1 |

**Supplementary Table S3.** Checklist for various newly developed assistive devices by job strain index.

| Job Strain Index | Intensity of exertion | Duration of exertion | Efforts/ minute | Hand/  wrist posture | Speed of work | Duration per day | Score |
| --- | --- | --- | --- | --- | --- | --- | --- |
| Bare hands | 6 | 2 | 1 | 3 | 1 | 1 | *36.0 |
| Sucker | 3 | 2 | 1 | 3 | 1 | 1 | *18.0 |
| Push-open | 2 | 1.5 | 1 | 1 | 1 | 1 | 3.0 |
| Rotate-open | 1 | 1.5 | 1 | 1 | 1 | 1 | 1.5 |
| Automatic cap opening device | 0 | 1.5 | 1 | 1 | 1 | 1 | 0.0 |

*Indicates danger if further improvements are not made
